# Effect of leukocyte-reduced platelet-rich plasma on osteoarthritis caused by cranial cruciate ligament rupture: A canine gait analysis model

**DOI:** 10.1371/journal.pone.0194752

**Published:** 2018-03-19

**Authors:** José M. Vilar, Maria E. Manera, Angelo Santana, Giuseppe Spinella, Oliver Rodriguez, Mónica Rubio, José M. Carrillo, Joaquín Sopena, Miguel Batista

**Affiliations:** 1 Departamento de Patología Animal, Facultad de Veterinaria de la Universidad de Las Palmas de Gran Canaria, Trasmontaña S/N, Arucas, Las Palmas, Spain; 2 Instituto Universitario de Investigaciones Biomédicas y Sanitarias, Universidad de Las Palmas de Gran Canaria, Juan de Quesada 30, Las Palmas de Gran Canaria, Las Palmas, Spain; 3 Dipartimento di Science Mediche Veterinarie, Facoltà di Veterinaria de la Università di Bologna, Via Tolara di sopra 50, Ozzano, Bologna, Italy; 4 Departamento de Medicina y Cirugía Animal, Universidad CEU Cardenal Herrera, C/ Tirant lo Blanc, 7, Alfara del Patriarca, Valencia, Spain; Illinois State Museum, UNITED STATES

## Abstract

The goal of this study was to objectively assess the effect of a platelet-rich plasma (PRP) derivate in English bulldogs with stifle degenerative joint disease secondary to cranial cruciate ligament rupture (CCLR). We used a force platform and affixed electrogoniometers to measure peak vertical force (PVF), vertical impulse (VI), stance time (ST), and angular range of motion (AROM), from 12 lame client-owned English bulldogs with post-CCLR stifle joint abnormalities. The 12 affected subjects were treated with 4 intra-articular injections of PRP, at 30-day intervals. Ten untreated, sound English bulldogs were used as a reference group. Clinical outcomes were evaluated using a linear mixed effects model. Mean values of PVF, VI, ST, and AROM were improved within the first 3 months post-treatment in the CCLR group, with mean measured changes increasing to maximum 4.56% body weight gain, 1.5% body weight/second, 0.07 seconds, and 6.18 degrees, respectively. The effects declined progressively after the treatment interval, ending at nearly initial levels after 6 months. This study demonstrates that dogs with CCLR treated with intra-articular PRP had improved PVF, VI, ST, and AROM over time; the duration of effect was waning by the end of the post-treatment period.

## Introduction

Cranial cruciate ligament rupture (CCLR) is one of the most significant stifle injuries in domestic dogs, and is a common cause of chronic lameness [1]. A CCLR results in joint instability leading to osteoarthritis (OA) and degenerative joint disease [2–6]. Progressive joint degeneration produces changes in catabolic and anabolic activity of chondrocytes [7], impairing functional capability, producing pain, limiting flexibility and motion and lowering quality of life [8].

Various surgical techniques have been advocated to treat CCLR, including extracapsular stabilization, tibial plateau-leveling osteotomy, and tibial tuberosity advancement [9]. Conservative management includes weight loss [10], omega-3 fatty acid supplementation [11] and use of NSAID drugs [12].

Recently, innovative treatments for modulating joint pain have been introduced, including chemical precursors, mesenchymal stem cell therapy, and growth factors obtained from platelet-rich plasma (PRP). These treatments can be used alone or in combinations [6,13]. Platelets have high concentration of growth factors, making PRP an appealing therapeutic alternative. The platelet growth factors could favorably influence need for analgesia, soft tissue healing, regulation of anti-inflammatory signals [14], and vascularizing and innervating of autografts [15].

Based on experimental results, PRP alone or as a co-therapy with surgical repair, can reduce progression of OA, without undesirable side effects [16,17]. It can be difficult to compare therapeutic studies directly because of differing assessment criteria, clinical measures (ie, OA classification systems), inclusion and exclusion criteria, varying aetiologies or the many possible intrinsic patient factors. Thus, objective measures of level and duration of efficacy represent significant advancements in experimental methods [18].

Force platform gait analysis is an objective, quantitative, non-invasive, and reliable method to characterize ground reaction forces during locomotion [19,20]. This kinetic methodology has been used to characterize gait in dogs after CCLR [1], but not for objective assessment of the effect of PRP in dogs with stifle OA that is secondary to naturally-occurring CCLR. On the other hand, electrogoniometers have been used to measure dog joint metrics such as range of angular movement (AROM) [21].

We hypothesize that PRP therapy has beneficial effects in dogs with CCLR, but that this effect may be limited in terms of time. To test this hypothesis, we used force platform and electrogoniometric gait analysis to determine efficacy and duration of effect of PRP therapy in English bulldogs with unilateral CCLR. An additional goal was to estimate an initial dosing regimen for further research.

## Materials and methods

### Dogs

All procedures were approved by the Ethics Committee of the Research Institute in Biomedical and Health Sciences of the University of Las Palmas de Gran Canaria (Spain), in compliance with national (Royal Decree 1201/2005) and European Union regulations (European Directive 86/609/CE) for projects using animals for research. A total of 22 adult, client-owned English bulldogs were included in the study. Ten of them served as clinically normal, unaffected, untreated control group. General inclusion criteria consisted of body weight between 18–27 kg and age between 1.5–6 years.

#### CCLR group

To qualify for our study design, post-CCLR dogs (n = 12) would be overtly lame on the ipsilateral injury side and would lack evidence of concurrent systemic or other orthopaedic disease. Medical screening included hematology, urinalysis, serum biochemical profiles, and no medication given over the preceding 4 weeks. All dogs were evaluated for articular effusion and for tibial compression and cranial drawer tests to assess the integrity of the cranial cruciate ligament.

Bilateral stifle radiographs were made under tibial compression with each dog in ipsilateral recumbency. CCLR was confirmed by observing cranial displacement of the proximal end of the tibia with respect to the femoral condyles, and any radiographic signs that were consistent with osteoarthritis. The radiological and physical evaluations also were needed to confirm that the stifle joint disease was unilateral.

#### Control group

The dogs selected for the Control group (n = 10) would not have current or previous history of orthopaedic or neurologic disease, nor persistent injury or systemic disease, confirmed by general physical examination, orthopedic evaluation, and radiology. None of the control dogs were forced to perform physical activities. Dog owners signed an informed consent form that allowed their dogs to participate in the study.

### Preparation and administration of PRP

Prior to platelet collection and activation, blood platelet and leukocyte counts were obtained. Whole blood (10 mL) was extracted aseptically from a cephalic vein and collected in two 5-mL centrifuge tubes, each containing 1.0 mL sodium citrate (2.5% solution) as anticoagulant. The tubes were centrifuged (Centronic JP selecta, model 7000015, Barcelona, Spain) for 10 min at 210 x *g*. Only the lower one-third of the yielded plasma (adjacent to the buffy coat) was activated with 5% of its volume, using 10% calcium chloride. The resultant ~2 mL solution was injected aseptically into the affected stifle joint through the conventional arthrocentesis site [22]. The appearance of joint fluid confirmed proper needle placement. A total of four doses were administered on days 0, 7, 14 and 21. After each inoculation, exercise was restricted for 2 days by confining to a walk at a maximum of 30 minutes/day on a leash. For the remaining days, dogs could walk on a leash a maximum of three times daily for 30 minutes, until the end of the experiment. No dogs were engaged in strenuous activity during the study.

### Gait analysis

Kinetic analysis was performed using a single platform mounted in the centre of, and level with, a 7-m runway covered by a rubber mat. The mat weight was eliminated by setting it to “0 force” with the tare button, after the platform was covered. Dogs were leash-guided at a walk over the force platform, always by the same handler. Walk velocity was measured using a motion sensor (Pasco, Roseville, California, USA) positioned 1-m from the platform.

Five valid trials were obtained for each dog, at a sampling frequency of 250 Hz. A trial was considered valid when the limb fully contacted the force platform and the dog walked next to the handler without pulling on the leash. The trial was discarded if the dog was distracted during the measurement, if the limb struck the edge of the force plate, or if any portion of the contralateral paw hit the force plate. A member of the research team (OR) evaluated the trial to confirm which limb touched the centre of the force platform. The platform was interfaced with a dedicated computer using DataStudio (Pasco, Roseville, California, USA), a software specially designed for acquisition, numerical conversion, and storage of data. A team member (JMV) recorded data from affected limbs at Day D0, D30, D90 and D180 after the first administration of PRP. Data from sound dogs were obtained at the same intervals; the Peak Vertical Forces (PVF) and Vertical Impulse (VI) values were normalized to body weight (as %) to characterize possible improvement of lameness during treatment with PRP. The Stance Time (ST) value was acquired directly from DataStudio.

Immediately after the force platform examination, electrogoniometry was performed using an electrogoniometer (Pasco) placed over the lateral collateral ligament of the stifle and affixed with adhesive plasters by an experienced researcher (OR). After the dogs were observed to be comfortable with the device, five consecutive steps were recorded. Electrogoniograms were obtained with the same software used to obtain the AROM.

### Statistical analysis

The purpose of the experiment was to determine if systematic differences exist in PVF, VI, ST and AROM in dogs with CCLR under PRP treatment given at four fixed moments in time: D0, D30, D90, D180. For this reason, a linear mixed effects model was used with the experimental factor (time) as a fixed effect and the blocking factor (dog) as a random effect. The model is as follows:

$$y_{ijk}=\mu_{j}+b_{i}+b_{ij}+\mu_{ijk};\;i=1,\ldots ,5;\;j=1,\ldots ,4;\;k=1,\ldots ,n_{ij}$$

$$b_{i}\approx N(0,\sigma_{b});\;b_{ij}\approx N(0,\sigma_{l});\;\mu_{ijk}\approx N(0,\sigma )$$

where *y*_*ijk*_ is the *k-th* measure of PVF, VI, ST and AROM, for the dog (*i* = 1 … 5) on day *j* = 1 (0), 2 (30), 3 (90), and 4 (180); *μ*_j_ is the (fixed) effect of time *j*. This parameter represents PVF, VI, ST, and AROM in the CCLR group, at the *j-th* observation. For *b*_*i*_, the (random) effect of dog, values are anticipated to be normally distributed, with mean 0 and standard deviation *σ*_*b*_. Thus, *σ*_*b*_ is the variability in the response due to the dogs; is the interaction effect between dog and time of observation (*j*). This term allows for the possibility that some dogs could improve at the same time period as others worsen. μ_*ijk*_ is the residual in the measure *ijk*. This variable also is assumed to be normally distributed with mean 0 and standard deviation *σ*. *η*_*ij*_ is the number of replicates of the PVF, VI, ST and AROM, measures on the dog at each time interval.

Metrics in this model were estimated using the nlme package in **R** statistical software (https://www.r-project.org/). For comparing fixed effects, the multiple comparison test of Tukey was applied. For assessing the validity of the model, a Shapiro-Wilk test was applied for testing normality of the residuals, and a Levene’s test was done for testing homoscedasticity. Ninety-five percent confidence intervals for differences between CCLR and Control groups at serial intervals also were computed. Statistical power analysis was calculated with Glimmpse© (http://glimmpse.samplesizeshop.org/#/).

## Results

The CCLR group contained 4 female and 8 male dogs, and the Control group consisted of 4 female and 6 male dogs. The mean (± SD) body weight of enrolled dogs was 22.83 ± 2.62 kg in the CCLR group and 23 ± 3.01 kg in the Control group (P = 0.30). Mean age was 3.6 ± 1.14 in the CCLR group and 4 ± 1.22 in the Control group (P = 0.54).

The mean value for walking velocity of both sound (Control) and CCLR group dogs was 0.7 ± 0.2 m/s (P = 0.12). Mean values (± SD) for PVF, VI, ST and AROM, in both CCLR and Control groups are summarized in Table 1. Data were distributed normally and were homoscedastic.

**Table 1:** Mean and standard deviation (mean ± SD) of force plate analysis data (n = 22) for the lame leg in CCLR group and Control group. Data are shown in percent of dog weight (PVF, VI), in seconds (ST), and degrees (AROM).

Parameter	Day after treatment					
	D0	D30	D90	D180	SWT	LT
PVF					0.09	0.7
CCLR	35.13 ± 1.08#	39.69 ± 1.10*	38.47 ± 1.31*	36.10 ± 0.78#*		
Control	40.09 ± 0.84	39.34 ± 0.66	39.32 ± 0.76	39.37 ± 0.51		
VI					0.20	0.40
CCLR	10.79 ± 0.61#	13.28 ± 0.66*	11.69 ± 0.55*	11.19 ± 0.62#*		
Control	11.90 ± 0.60	11.89 ± 0.65	11.95 ± 0.58	11.82 ± 0.46		
ST					0.37	0.60
CCLR	0.38 ± 0.03#	0.45 ± 0.05*	0.43 ± 0.03#*	0.40 ± 0.03#*		
Control	0.43 ± 0.03	0.44 ± 0.04	0.45 ± 0.03	0.43 ± 0.03		
AROM					0.82	0.45
CCLR	25.10 ± 3.15#	30.78 ± 1.96*	31.25 ± 1.56*	25.49 ± 3.67#		
Control	31.58 ± 0.61	31.51 ± 0.62	31.62 ± 0.69	31.67 ± 0.58		

PVF = peak vertical force, CCLR = cranial cruciate ligament rupture group, VI = vertical impulse, ST = stance time, AROM = angular range of motion

# significant difference (p<0.05) between study and Control group.

* significant difference (p<0.05) in the study group between checking periods and D0, SWT Shapiro-Wilk test value, LT Levene test value.

The PRP obtained under this protocol revealed mean platelet count of 1013 ± 431 x 103 cells/μL (≈ 336% baseline count) and mean leukocyte count of 0.1 ± 0.1 x 103 cells/μL (Table 2).

**Table 2:** Weight and platelet count for each dog at D0, D30, D90 and D180.

	Weight	Day after treatment			
		D0	D30	D90	D180
CCLR					
Dog 1	19	284	296	270	280
Dog 2	21	331	314	314	313
Dog 3	23	458	488	410	435
Dog 4	25	248	232	253	237
Dog 5	27	367	371	385	342
Dog 6	18	418	423	421	417
Dog 7	23	295	297	293	297
Dog 8	24	318	323	317	323
Dog 9	26	416	423	418	421
Dog 10	23	273	275	278	277
Dog 11	23	346	351	342	353
Dog 12	22	375	384	383	387
CONTROL					
Dog 1	23	345			
Dog 2	19	464			
Dog 3	26	332			
Dog 4	21	222			
Dog 5	18	337			
Dog 6	24	416			
Dog 7	27	384			
Dog 8	22	295			
Dog 9	24	323			
Dog 10	26	269			

Units; weight: kg, platelets count: 103/μl

### Analysis of PVF

Comparing with D0, mean PVF in the CCLR group increased by 4.56% at D30 (P < 0.0001); 3.34% increase was found at D90 (P < 0.0001); and 0.97% increase at D180 (P = 0.0077).

**Fig 1:**
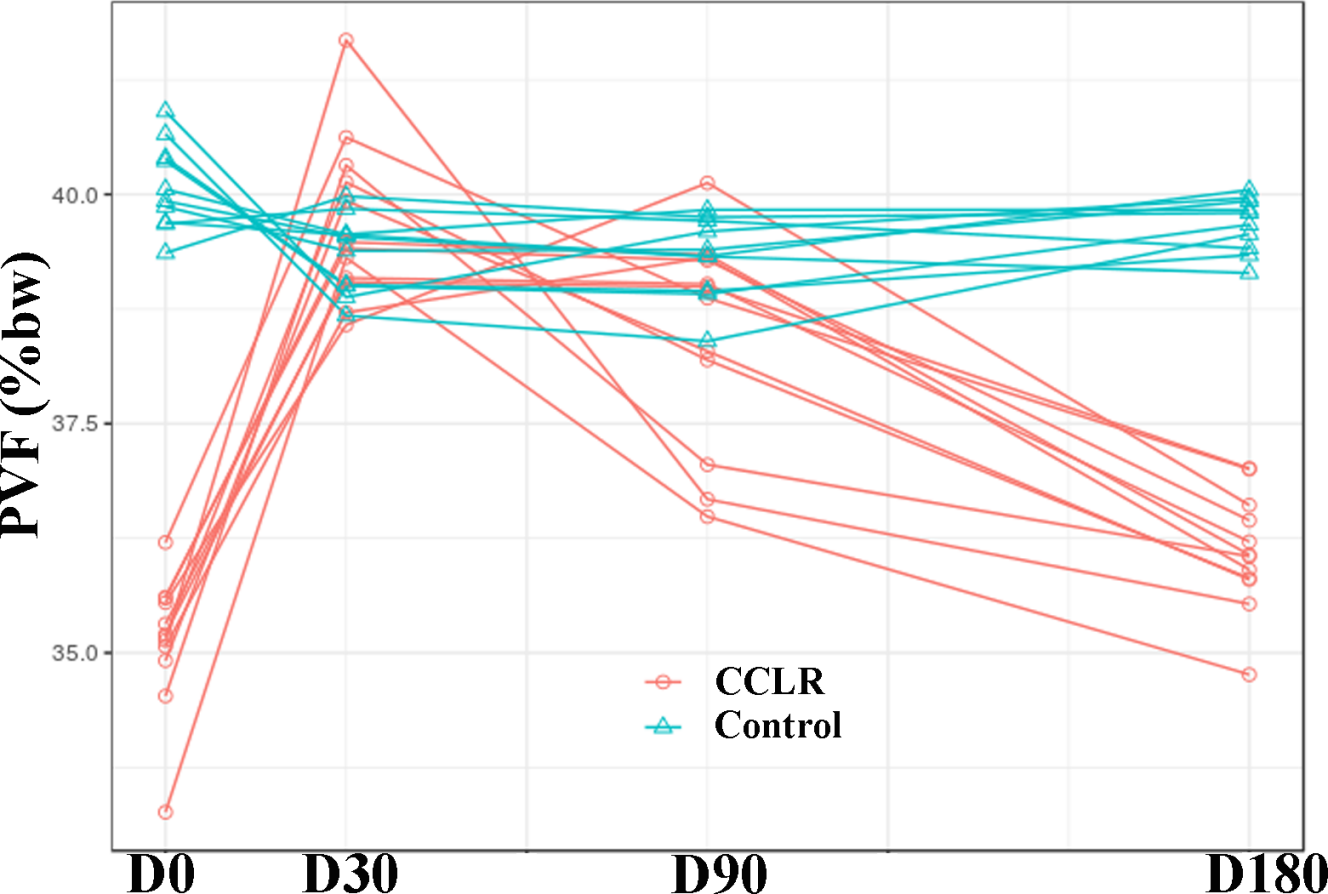
PVF values in CCLR group at the 6-month follow-up period after treatment with platelet-rich plasma. Data from Control group also are shown.

Compared with the Control group, lower PVF was found at D0 (4.96%) (P < 0.0001) and D180 (3.57%) (P < 0.0001) (Fig 1).

### Analysis of VI

VI was increased 1.5% at D30 (P < 0.0001), 0.9% at D90 (P < 0.0001), and 0.4% at D180, all as compared with day 0 (P = 0.003).

**Fig 2:**
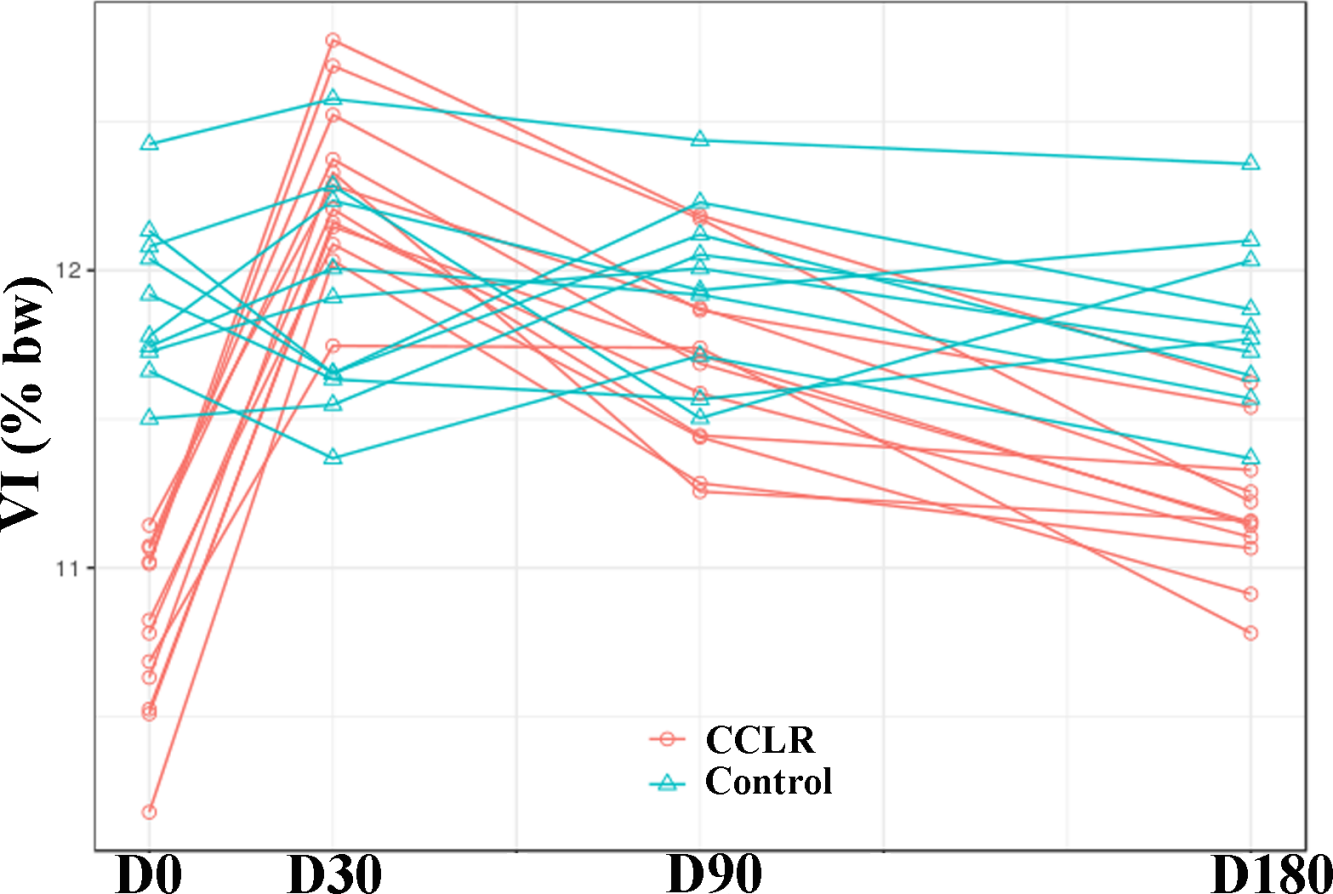
VI values in CCLR group at the 6-month follow-up period after treatment with platelet-rich plasma. Data from Control group also are shown.

Comparison of the CCLR and Control groups revealed significant differences at D0 (1.13% less) (P < 0.0001), and at D180 (0.63% less) (Fig 2) (P < 0.0001).

### Analysis of ST

In the CCLR group, with respect to D0, ST increased in 0.07s, 0.04s, and 0.03s at D30 (P = 0), D90 (P = 0), and D180 (P = 0.004224), respectively.

**Fig 3:**
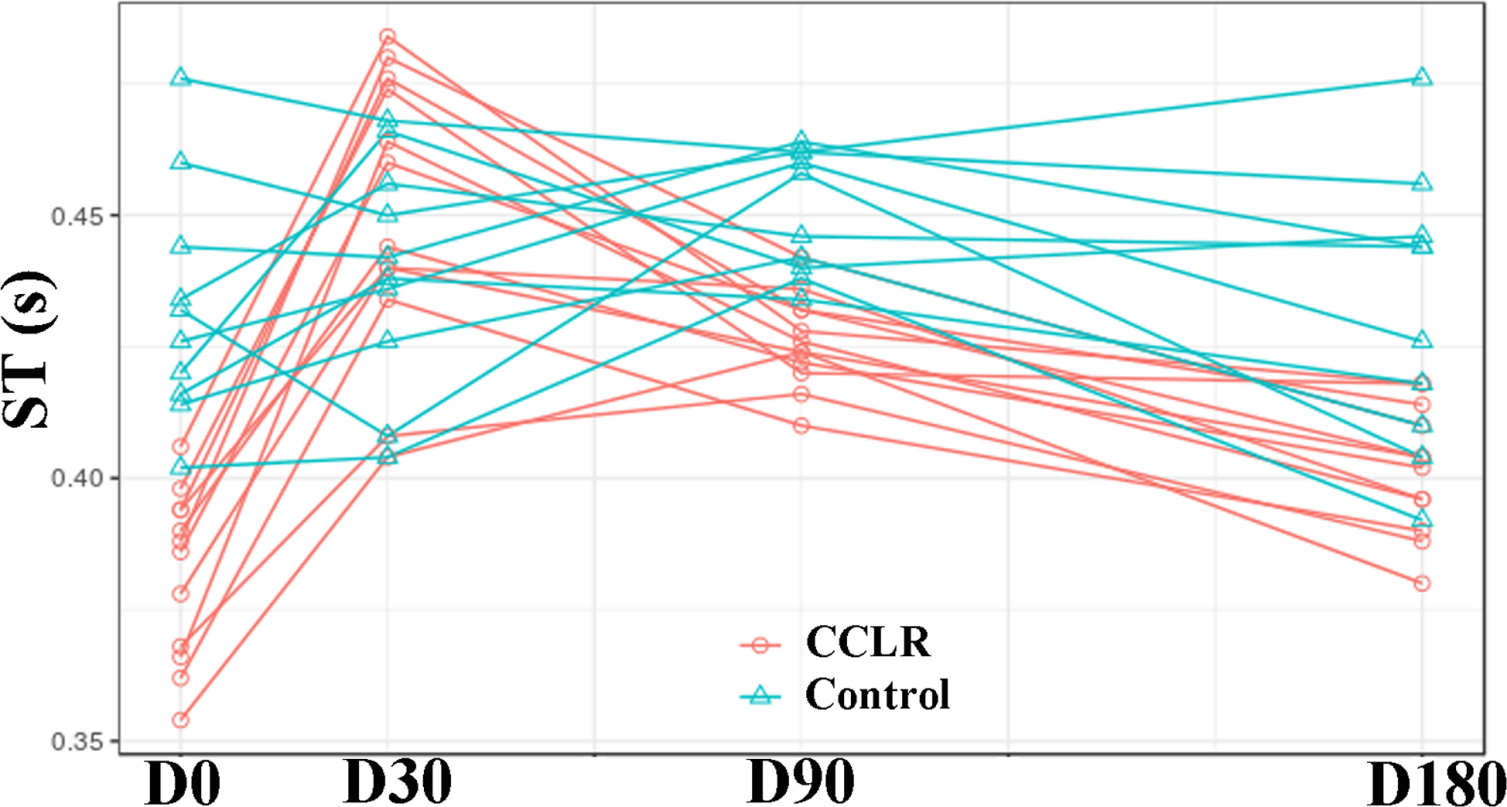
ST values in CCLR group at the 6-month follow-up period after treatment with platelet-rich plasma. Data from Control group also are shown.

Compared with Controls, affected dogs’ ST was 0.05s less at D0 (P = 9.1–9). At D30, the difference was not significant. At D90 and D180, ST again was lower (0.044s and 0.019s, respectively) (P = 0.039) (Fig 3).

### Analysis of AROM

Analysis of variance revealed that at D30, AROM increased by 5.68 degrees, compared with D0 (P < 0.0001). AROM increased to 6.18 degrees at D90 (P < 0.0001).

**Fig 4:**
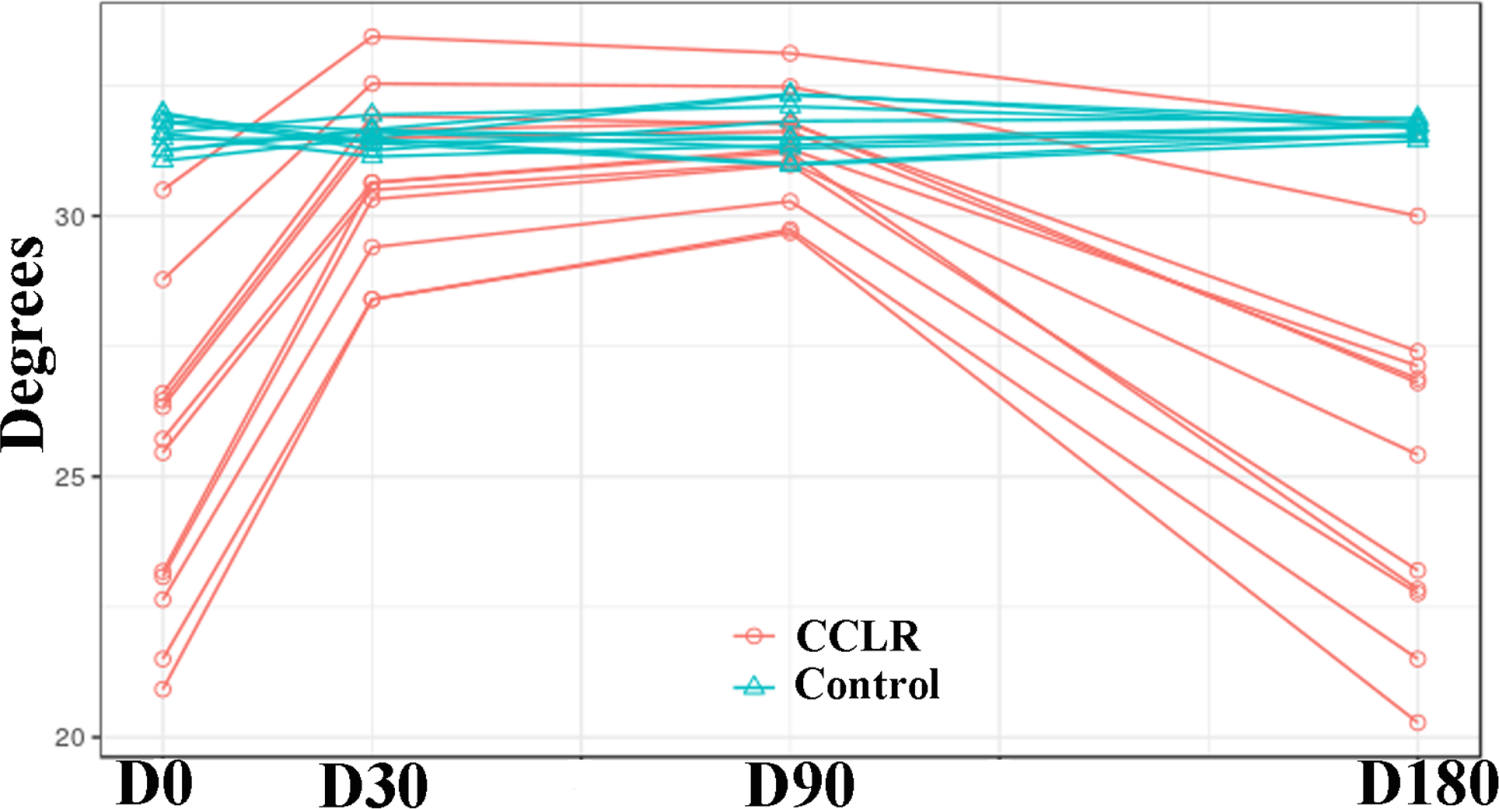
AROM values in CCLR group at the 6-month follow-up period after treatment with platelet-rich plasma. Data from Control group also are shown.

Compared with the Control group, CCLR dogs had AROM 6.48 degrees less at D0 (P < 0.0001); at D30 and D90, no differences were found. However, at D180, AROM decreased by 6.17 degrees (P < 0.0001) (Fig 4).

Our sample size of 12 dogs provided a statistical power of 83% for the detection of differences of 0.5% of each parameter among individuals.

## Discussion

We assessed PVF, VI, ST, and AROM changes in lame English bulldogs with unilateral CCLR treated with PRP. We used a force platform and electrogoniometry. The data provided support our hypothesis that PRP therapy is effective for OA secondary to CCLR, although the effect is temporary.

To our knowledge, this is the first study that provides objective data regarding PRP treatment outcomes in dogs with sequelae to naturally occurring CCLR.

PRP therapy was demonstrated to have a positive effect on limb function. Specifically, we observed improved locomotion, associated with reducing both lameness and gait disability, allowing the dogs to support more weight on their hind limbs and reach greater AROM.

Important factors that make it difficult to compare results among studies are the diverse methods and devices used to evaluate pain and lameness. In this sense, PRP joint therapy requires more investigation, focused specially on assessing its efficacy across therapies, using objective, and reliable assessment tools.

When kinetic evaluation is performed, some authors state that the force plate gait analysis yields large variation in normal ground reaction forces, preventing meaningful direct comparison of data among different dogs or among dog groups having differing conformation (breed) [23]. Under this premise, comparative studies necessarily require rescaling, harmonization, or normalization of data. In our study, we chose all dogs of the same breed to provide more standardized data sets.

Regarding ST, a kinematic parameter, one report indicated that a group of dogs treated with NSAIDs experienced decrease in ST when they were able to improve their performance [24]. However, other reports indicate that ST remained unchanged or increased as limb function improved [25,26]. If pain is suppressed effectively, we suggest that ST should improve after effective treatment.

Recent reports have shown that the presence of leukocytes in PRP preparations may be detrimental to healing, due to their involvement in the inflammatory response [27] causing loss of pain relief efficacy [28]. We avoided this complication, because our PRP was prepared with nearly complete absence of leukocytes.

Concerning actual administration, there is no consensus at this time about a standard regimen. Thus, PRP treatment for OA can vary from one to a series of nine injections [29–33]. Under this range of treatment protocols, we decide to apply a mid-level of four injections at 7-day interval.

A follow-up evaluation of 6 months could be considered as a minimum standard for testing the outcome of a medical or surgical treatment. In our study, this time was sufficient to recognize significant improvement during the first months after treatment, and also to establish that lame dogs eventually returned nearly to their initial status. The duration of the effect that we noted is consistent with previous reports [34,35]. Based on our results and taking into account that improvement by surgical means can extend relief for more than 2 years [29], PRP therapy may become an alternative resource for those instances wherein affected dogs cannot undergo surgery because of anaesthetic risk or when dog owners cannot afford surgical costs. Other commercially available PRP derivates (PRGF- Endoret®, Orthokine®) have been associated with better effect and/or longer duration, but objectively-generated data remain to be collected, as is the case also with injections of hyaluronic acid or NSAIDs.

Our study has some limitations. First, the number of available dogs was relatively small. Small sample sizes can associate with low statistical power, and previous reports have established that dog conformation and size can affect kinetic measures [23]. Therefore, we decided to prioritize data homogeneity (diminishing the inter-subject variability) by using dogs from the same breed within defined weights and ages. We thus noted that variation of only 0.5% in PVF could be detected with a statistical power of 83%.

Second, although both hind limbs were recorded, we provide only data from the affected limbs for comparison with control dogs. Based on previous reports, force redistribution to contralateral limbs and even to forelimbs (in hind limb lameness) could call this choice into question [36].

Third, our study design included sound dogs as controls when, typically, a Control group would have included untreated affected dogs. However, painful untreated or placebo-treated dogs in a positive Control group could worsen, rendering these dogs unable to provide fixed reference data. Additionally, ethical concerns arise when affected, untreated controls are part of biomedical experimental designs. In accordance with other authors using similar study designs [23], data from sound dogs were provided as an “ideal status” disease-free Control group.

## Conclusion

Force platform and electrogoniometric gait analysis established quantitatively that PRP therapy could be useful for treatment of chronic lameness in post-CCLR dogs. Our data indicate efficacy over a period of 3–6 months, without side effects. Maintaining efficacy for durations longer than 3–6 months requires further research.

## Supporting information

S1 File Datasets. (CSV)
